# Positron Emission Tomography With ^18^F-Fluorodeoxyglucose in Patients With Sickle Cell Acute Chest Syndrome

**DOI:** 10.1097/MD.0000000000000821

**Published:** 2015-05-08

**Authors:** Nicolas de Prost, Myriam Sasanelli, Jean-François Deux, Anoosha Habibi, Keyvan Razazi, Frédéric Galactéros, Michel Meignan, Bernard Maître, Christian Brun-Buisson, Emmanuel Itti, Armand Mekontso Dessap

**Affiliations:** From the Assistance Publique-Hôpitaux de Paris (NP, KR, CB-B, AMD), Hôpitaux Universitaires Henri Mondor, DHU A-TVB, Service de Réanimation Médicale; UPEC-Université Paris-Est Créteil Val de Marne (NP, KR, CB-B, AMD), Faculté de Médecine de Créteil, CARMAS Research Group; UPEC-Université Paris-Est Créteil Val de Marne (MS, J-FD, AH, FG, MM, BM, EI), Faculté de Médecine de Créteil; Assistance Publique-Hôpitaux de Paris (MS, MM, EI), Hôpitaux Universitaires Henri Mondor, Service de Médecine Nucléaire; Assistance Publique-Hôpitaux de Paris (J-FD), Hôpitaux Universitaires Henri Mondor, Service de Radiologie; Assistance Publique-Hôpitaux de Paris (AH, FG), Hôpitaux Universitaires Henri Mondor, Unité des Maladies Génétiques du Globule Rouge – Service de Médecine Interne; and Assistance Publique-Hôpitaux de Paris (BM), Hôpitaux Universitaires Henri Mondor Antenne de Pneumologie, Service de Réanimation Médicale, Créteil, France.

## Abstract

The acute chest syndrome (ACS) is the main cause of mortality among adult patients with sickle cell disease (SCD). Its pathophysiology is still unclear. Using positron emission tomography (PET) with ^18^F-fluorodeoxyglucose [18F-fluorodeoxyglucose (^18^F-FDG)], we explored the relationship between regional lung density and lung metabolism, as a reflection of lung neutrophilic infiltration during ACS.

Patients were prospectively enrolled in a single-center study. Dual modality chest PET/computed tomography (CT) scans were performed, with ^18^F-FDG emission scans for quantification of regional ^18^F-FDG uptake and CT scans with radiocontrast agent to check for pulmonary artery thrombosis. Regional lung ^18^F-FDG uptake was quantified in ACS patients and in SCD patients without ACS (SCD non-ACS controls). Maximal (SUVmax) and mean (SUVmean) standardized uptake values were computed.

Seventeen patients with ACS (mean age 28.3 ± 6.4 years) were included. None died nor required invasive mechanical ventilation. The main lung opacity on CT scans was lower lobe consolidation. Lungs of patients with ACS exhibited higher SUVmax than those of SCD non-ACS controls (2.5 [2.1–2.9] vs 0.8 [0.6–1.0]; *P* < 0.0001). Regional SUVmax and SUVmean was higher in lower than in upper lobes of ACS patients (*P* < 0.001) with a significant correlation between lung density and SUVmax (*R*^2^ = 0.78). SUVmean was higher in upper lobes of ACS patients than in lungs of SCD non-ACS controls (*P* < 0.001). Patients with SUVmax >2.5 had longer intensive care unit (ICU) stay than others (7 [6–11] vs 4 [3–6] days; *P* = 0.016).

Lungs of patients with ACS exhibited higher ^18^F-FDG uptake than SCD non-ACS controls. Lung apices had normal aeration and lower ^18^F-FDG uptake than lung bases, but higher ^18^F-FDG uptake than lungs of SCD non-ACS controls. Patients with higher lung ^18^F-FDG uptake had longer ICU stay than others.

## INTRODUCTION

The acute chest syndrome (ACS) is the main cause of mortality among patients with sickle cell disease (SCD), one of the most frequent monogenic diseases. The presentation of ACS associates fever and respiratory symptoms (including cough, dyspnea/acute respiratory failure, and chest pain) to new pulmonary infiltrates. The pathophysiology of ACS is only partly understood and combines the following mechanisms: respiratory infections,^1^ pulmonary artery thrombosis,^2^ hypoventilation associated with intense chest wall pain, and pulmonary fat embolism in a context of osseous vasoocclusive crisis^3–5^ and environmental insults.^6^ Lung computed tomography (CT) scan studies recently depicted the following patterns as typical for ACS^7^: complete consolidation of dorsal regions (lower lobes), contrasting with normal aeration of ventral ones (upper lobes). The previous finding that bronchoalveolar lavage fluids of ACS patients showed lung neutrophilic infiltration suggests that these consolidated lung regions would be fraught with intense regional inflammation.^4,8^ However, the topographic distribution of lung inflammation during ACS and whether regions with increased lung density would exhibit more lung inflammation than normally aerated regions has never been studied.

Lung imaging with positron emission tomography (PET) using ^18^F-fluorodeoxyglucose (^18^F-FDG), a glucose analog that is predominantly taken up by metabolically active cells, allows for noninvasive quantification of lung metabolism. In the inflamed nontumoral lung, ^18^F-FDG accumulation has been shown to be a marker of lung neutrophilic inflammation^9–12^ and a valuable tool to study the mechanisms of the acute respiratory distress syndrome,^13–15^ predict the development of severe acute respiratory failure,^16^ and evaluate the effects of therapeutic interventions.^17^

We hypothesized that during ACS consolidated lung regions would exhibit greater lung metabolism, reflecting local recruitment of activated neutrophils; and that patients with higher lung metabolism would have a more severe course than others. We conducted a prospective study in patients with ACS aiming at studying the relationship between regional lung structure, as assessed by density, and lung metabolism, reflecting lung neutrophilic infiltration; and testing the hypothesis that patients with higher lung metabolism would exhibit poorer outcomes than others.

## METHODS

### Study Subjects

This prospective single-center study was approved by the institutional ethics committee (Comité de Protection des Personnes Ile de France IX) of Henri Mondor Hospital, Créteil, France, which hosts the National French SCD Referral Center, and written, informed consent was obtained from all subjects. Consecutive adults (≥18 years) with SCD and a severe ACS requiring admission to the medical intensive care unit (ICU)/intermediate care unit^18^ between January 2012 and August 2013 were included. Inclusion criteria were as follows: ACS since <72 hours, defined as a new pulmonary infiltrate on chest x-ray together with either a respiratory symptom (dyspnea or chest pain) or an abnormal sound on lung auscultation. Exclusion criteria were as follows: antibiotic treatment since >48 hours, pregnancy, obesity (body mass index > 30 kg/m^2^), lung cancer, invasive or noninvasive mechanical ventilation support, shock, blood glucose level >11 mmol/L within 24 hours of PET scanner, creatinine clearance <30 mL/min, or any contraindication to iodine agents.

All patients received a uniform standardized treatment protocol for ACS including rehydration, oxygen therapy, analgesia using controlled-release intravenous morphine, antibiotic treatment, and red blood cells (RBCs) transfusion or partial-exchange transfusion depending on admission hemoglobin level.^8^ Patients were categorized as having respiratory infection or not based on a comprehensive microbiological workup including blood and sputum cultures, urinary antigen tests for legionella and *Streptococcus pneumoniae,* respiratory virus detection by multiplex polymerase chain reaction from nasopharyngeal swab (influenza A and B, parainfluenza, adenovirus, rhinovirus, coronavirus, bocavirus, metapneumovirus, and respiratory syncytial virus), and paired serum antibodies testing for *Mycoplasma pneumoniae*, *Legionella pneumophila*, and *Chlamydia pneumoniae* infections. Patients were discharged from the ICU when the following criteria were met: decreasing dose of intravenous morphine required for relieving pain, nasal oxygen requirement <3 L/min, respiratory rate <25 /min, and absence of extrapulmonary organ failure. Clinical and laboratory findings were recorded prospectively on admission and along the hospital course using standardized case report forms.

### Images Acquisition and Analysis

Dual modality chest PET/computed tomography (CT) scans were performed within 24 hours of inclusion in the study. Patients were fasted and glucose or insulin-containing infusions were discontinued for at least 6 hours. They were then transported to the Nuclear Medicine Facility by the attending physician under cardiorespiratory monitoring while pursuing oxygen delivery and morphine analgesia. Two serial sets of images were acquired on the same camera (Gemini GXL16, Philips, Da Best, The Netherlands), which are as follows.

Chest PET/CT scans were obtained 45 minutes after intravenous administration of ^18^F-FDG (5 MBq/kg) for quantification of regional ^18^F-FDG uptake. A low-dose helical CT was first performed for anatomical correlation and attenuation correction from the neck to the upper abdomen with the following parameters: x-ray tube tension of 120 kV, current of 80–100 mAs, rotation time 0.5 s, pitch 0.938, and slice thickness 2 mm. Images were reconstructed using line of response-row action maximum likelihood algorithm (2 iterations, 28 subsets, postfilter 5.1 mm), with and without CT attenuation correction (matrix size of 128 × 128, voxel size 4 × 4 × 4 mm^3^). Emission images were then acquired using 3 to 4 bed positions of 2 minutes each. Images were analyzed by 2 staff members of the Nuclear Medicine Department (M.S. and E.I.) who had no access to the medical charts of the patients. Regional lung ^18^F-FDG uptake was quantified using the standardized uptake value (SUV)^19^ as follows: regions of interest (ROIs) were manually delineated using a simplification approach in each lower and upper lobe (ie, on both sides), excluding vessels, main bronchi, and pleura with CT images, after visual identification of the transaxial cross section wherein lung parenchyma exhibited the highest visual ^18^F-FDG uptake. ROIs were then superimposed on both CT and PET images and SUVmax and SUVmean were recorded in upper and lower lobes on both the sides, as well as in liver (hepatic SUVmax), as a reference tissue,^16,20^ together with the mean density of the ROIs, expressed in Hounsfield units (HU). The tissue fraction of each ROI was computed from mean HU values, as previously described^21^: 
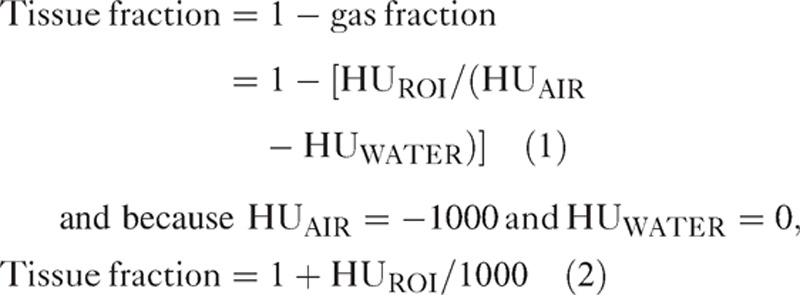


Chest spiral CT scan with radiocontrast agent was performed with the 16-row multidetector CT of the PET/CT camera, from the lung apices to the diaphragm. Contrast-enhanced CT was triggered by the bolus tracking technique, using an automatic, dual-head injector, with 100 mL of nonionic contrast medium (Iomeron 400; Bracco Imaging, Paris, France) injected at a rate of 3.5 mL/s via a 18-gauge peripheral intravenous catheter inserted in the antecubital vein or a central catheter, followed by a 40 mL saline flush, with bolus tracking. CT parameters were as follows: x-ray tube tension of 120 kV, current of 250 mAs, rotation time 0.5 s, pitch 0.938, and slice thickness 2 mm. The median CT volume dose index was 721 mGy.

Images were analyzed by 1 staff member of the Radiology Department (J.F.D.) who had no access to the medical chart of the patients. Lung opacities were defined according to the Fleischner Society Glossary of Terms for Thoracic Imaging.^7,22^ Briefly, a consolidation was defined as a homogeneous increase in pulmonary parenchymal attenuation that obscured the margins of vessels and airway walls (an air bronchogram might be present). Atelectasis was defined by a reduced volume, accompanied by attenuation in the affected part of the lung. Ground-glass opacity was defined as hazy increased opacity of lung, with preservation of bronchial and vascular margins. Pulmonary artery thrombosis was defined as a thrombus up to the segmental level or multiple thrombi at the subsegmental level. For each type of lung opacity (consolidation, ground glass, and atelectasis), the radiologist rated the number of segments involved in each lung lobe.

### SCD Non-ACS Controls

Seven age-matched patients (median [range] age: 26 years [23–49]) with SCD (SS disease, n = 6; SC disease, n = 1), including 2 females and 5 males, underwent 10 PET/CT scans while having no ACS criteria and normal chest radiograph and were used as controls. Four of them had a previous history of ACS, 2 of them were following a chronic transfusion program and 1 of them received hydroxyurea. These patients had been fasted and glucose or insulin-containing infusions had been discontinued for at least 6 hours. Reasons for PET/CT were as follows: osteomyelitis (n = 2), suspicion or follow-up of extrathoracic malignancy (n = 6), and endocarditis (n = 2). ^18^F-FDG uptake (SUVmax and SUVmean) was measured in normally aerated lung regions of each side and in the liver. Three of these SCD patients had osseous vasoocclusive crises within 3 months of the PET/CT scans but none developed ACS.

### Statistical Analysis

The data were analyzed using Graphpad Prism version 5.00 for Windows statistical software (GraphPad Software, San Diego, CA; www.graphpad.com). Categorical variables, expressed as percentages, were compared using the χ^2^ test or Fisher exact test, as appropriate. Continuous data are expressed as mean ± standard deviation (if normally distributed) or as median (interquartile range 25%–75%) and were compared using the Student *t* test or the Mann–Whitney test for independent samples and the paired Student *t* test or Wilcoxon test for related samples, as appropriate. The parametric distribution of data was assessed using the Shapiro–Wilk normality test. Physiological and outcome variables were compared between patients with higher and those with lower lung SUVmax, using the median lung SUVmax as cutoff value (≤2.5 or >2.5) because no previous value of lung SUV was reported during ACS and because SUVmax is the most widely used parameter for quantifying ^18^F-FDG uptake in the clinical setting.^16,19,23^ The relationship between regional lung ^18^F-FDG uptake (SUVmax) and lung density (HUs) was assessed using both linear and nonlinear regression models, and coefficients of determination (*R*^2^) were compared to identify the model that fitted best the data. PET-acquired data were also compared using 2-way analysis of variance for repeated measures. Bonferroni corrected post-hoc tests were performed when overall *P* value was <0.05. Two-sided *P* values <0.05 were considered significant.

## RESULTS

Thirty-two patients were admitted in our ICU for ACS during the study period, including 15 with exclusion criteria (antibiotic therapy since >48 hours, n = 7; unavailability of the PET camera, n = 3; noninvasive ventilation support, n = 2; refusal to consent, n = 3). Seventeen patients were thus included in the study, including 16 with SS disease and 1 with S-β+ thalassemia disease.

### Lung CT Scans Findings

**FIGURE 1:**
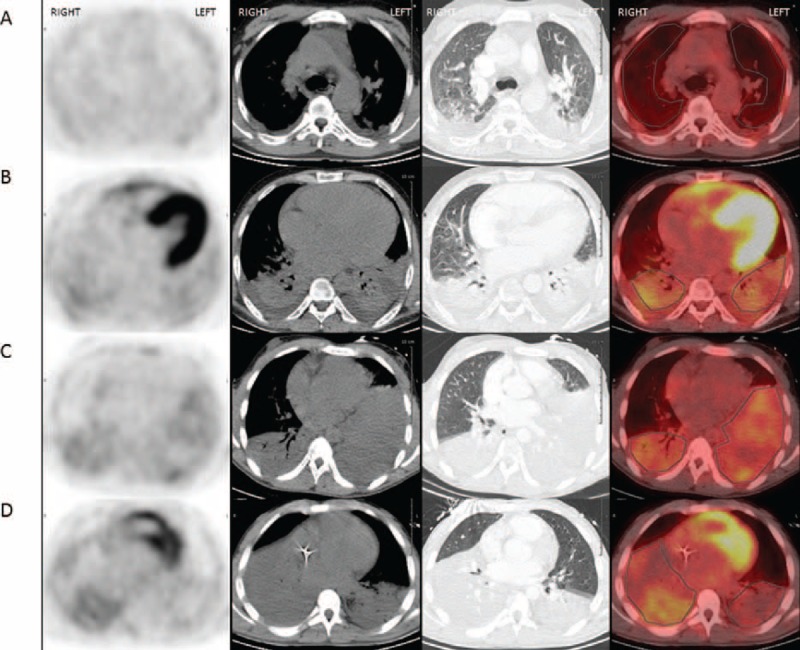
Examples of transaxial cross-section ^18^F-FDG PET CT images from 3 patients with sickle cell acute chest syndrome. From left to right, PET scan, CT scan with mediastinal and parenchymal windows, and PET CT fusion image. Cross-section images show low ^18^F-FDG uptake in normally aerated upper lobes (A) and higher ^18^F-FDG uptake in consolidated lower lobes (B) of the same patient (a bilateral pleural effusion is not included in the ROIs) and high ^18^F-FDG uptake in consolidated lower lobes of 2 distinct patients (C, D) with normally aerated adjacent lung parenchyma. CT = computed tomography, ^18^F-FDG = ^18^F-fluorodeoxyglucose, PET = positron emission tomography, ROIs = regions of interest.

The predominant lung opacity observed in patients with ACS was consolidation, located mainly in lower lobes, contrasting with a normal aeration of most of upper lung segments (Table 1, Figure 1). Ground-glass opacities were rare, with no lobar predominance, and atelectasis was almost never observed. A pulmonary artery thrombosis was found in 2 patients. Pleural effusion was found in 13 patients, including 3 cases with unilateral effusion and 10 cases with bilateral effusion. None of the patients had pulmonary fibrosis.

**TABLE 1:**
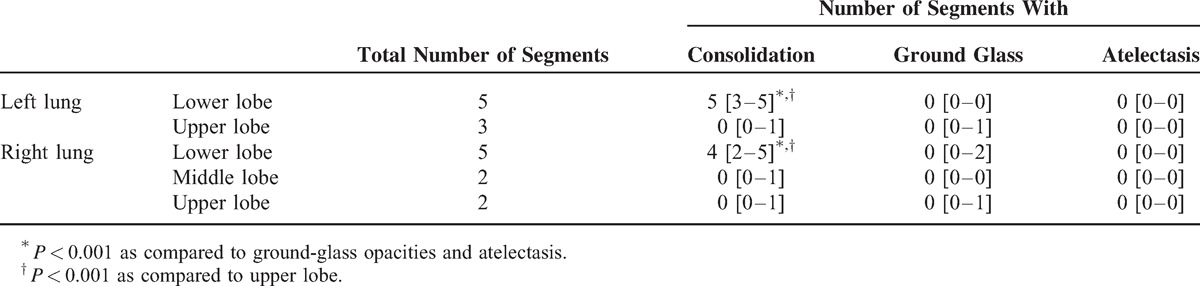
Distribution of Lung Opacities on Computed Tomography Scan in Patients With Acute Chest Syndrome

**FIGURE 2:**
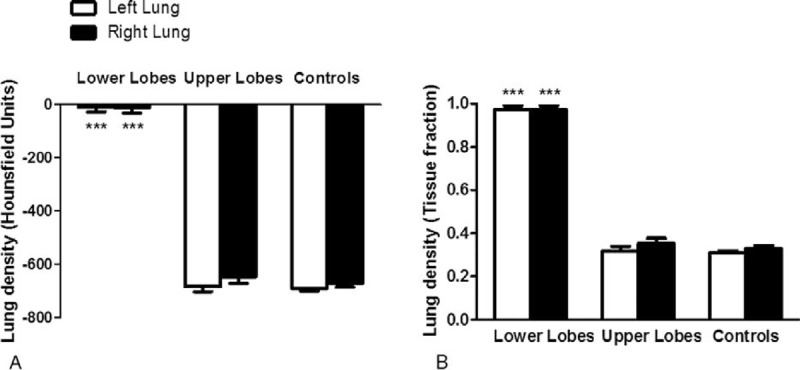
Lung density in patients with ACS and SCD non-ACS controls. HU (A) and tissue fraction (B) were consistent with consolidation of lung tissue in ROIs of lower lobes, whereas ROIs of upper lobes and lungs of SCD non-ACS controls showed normal aeration. Comparison using 2-way analysis of variance with repeated measures, with Bonferroni adjustments for multiple comparisons, showed a significant effect of lobes (*P* < 0.0001), but no significant effect of lung side (*P* = 0.31 for HU; *P* = 0.28 for tissue fraction) nor interaction (*P* = 0.62 for HU; *P* = 0.61 for tissue fraction). ^∗∗∗^*P* < 0.001, as compared to upper lobes and SCD non-ACS controls. ACS = acute chest syndrome, HU = Hounsfield units, ROIs = regions of interest, SCD = sickle cell disease.

Lung densities in ROIs drawn in lower lobes were consistent with those of consolidated lung tissue, with mean HU values close to zero and tissue fraction values close to 1 (Figure 2). In contrast, lung densities in ROIs of upper lobes were consistent with those of normally aerated lung tissue and not different from those of SCD non-ACS controls.

### ^18^F-FDG PET Scans Findings

**FIGURE 3:**
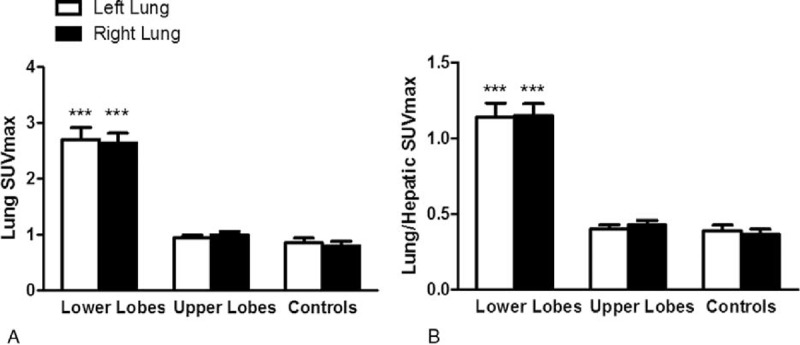
Lung maximal standardized uptake value (SUVmax) without (A) and with (B) normalization for hepatic SUVmax. Lower lobes of patients with ACS exhibited higher SUVmax, as compared with upper lobes and lungs of SCD non-ACS controls. Comparison using 2-way analysis of variance with repeated measures, with Bonferroni adjustments for multiple comparisons, showed a significant effect of lobes (*P* < 0.0001), but no significant effect of lung side (*P* = 0.86 and 0.87 without (A) and with (B) normalization, respectively) nor interaction (*P* = 0.80 and 0.79 without (A) and with (B) normalization, respectively). ^∗∗∗^*P* < 0.001, as compared to upper lobes and SCD non-ACS controls. ACS = acute chest syndrome, SCD = sickle cell disease.

**FIGURE 4:**
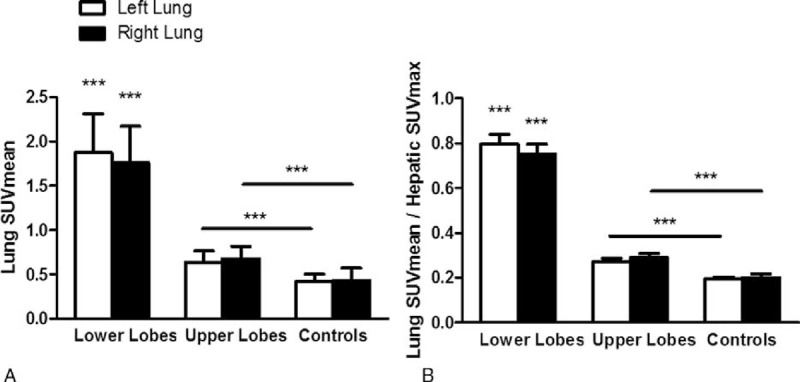
Lung mean standardized uptake (SUVmean) without (A) and with (B) normalization for hepatic maximal standardized uptake (SUVmax). Lower lobes of patients with ACS exhibited higher SUVmean, as compared to upper lobes and lungs of SCD non-ACS controls, and upper lobes exhibited higher SUVmean than lungs of SCD non-ACS controls. Comparison using 2-way analysis of variance with repeated measures, with Bonferroni adjustments for multiple comparisons, showed a significant effect of lobes (*P* < 0.0001), but no significant effect of lung side (*P* = 0.68 and 0.72 without (A) and with (B) normalization, respectively) nor interaction (*P* = 0.39 and 0.37 without (A) and with (B) normalization, respectively). ^∗∗∗^*P* < 0.001, as compared to upper lobes and SCD non-ACS controls or as indicated. ACS = acute chest syndrome, ROIs = regions of interest, SCD = sickle cell disease.

Lungs of patients with ACS exhibited higher ^18^F-FDG uptake than those of SCD non-ACS controls (SUVmax of 2.5 [2.1–2.9] vs 0.8 [0.6–1.0]; *P* < 0.0001). Regional ^18^F-FDG uptake, as assessed both visually and with SUVmax quantification, with and without normalization for hepatic SUVmax, was markedly higher in ROIs of lower lobes than in those of upper lobes (Figure 3). Computing SUVmean confirmed the previous finding of higher ^18^F-FDG uptake in ROIs of lower versus upper lobes; it also showed that ROIs of normally aerated upper lobes of ACS patients displayed significantly higher ^18^F-FDG uptake than lungs of SCD non-ACS controls (Figure 4). This finding highlights that in spite of showing normal lung density, upper lobes of ACS patients exhibited higher metabolism than those of patients with SCD but no current ACS. Results of ^18^F-FDG uptake parameters in patients with SUVmax >2.5 and ≤2.5 are presented in Table 2.

**TABLE 2:**
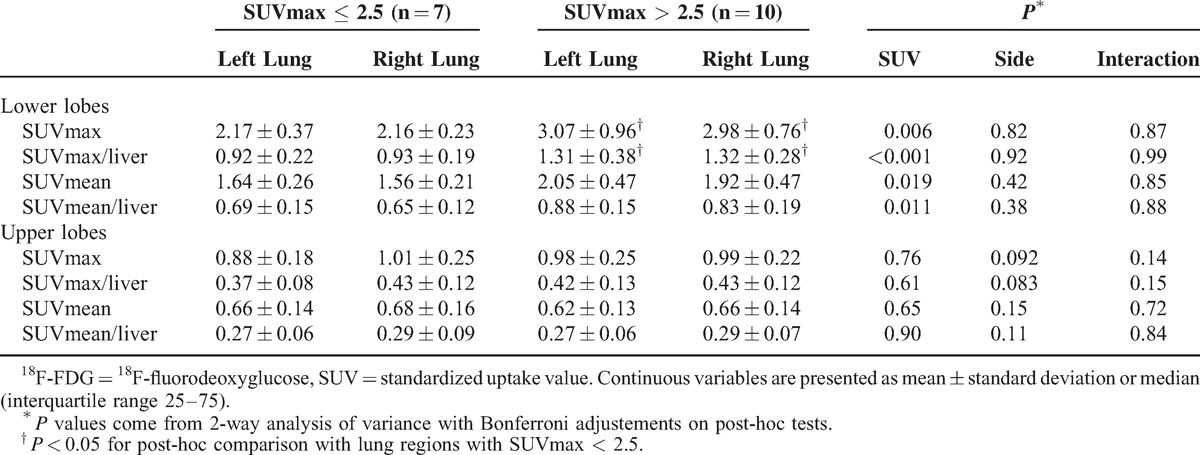
Lung ^18^F-FDG Uptake Parameters of Patients With Acute Chest Syndrome According to Maximal SUV Values

**FIGURE 5:**
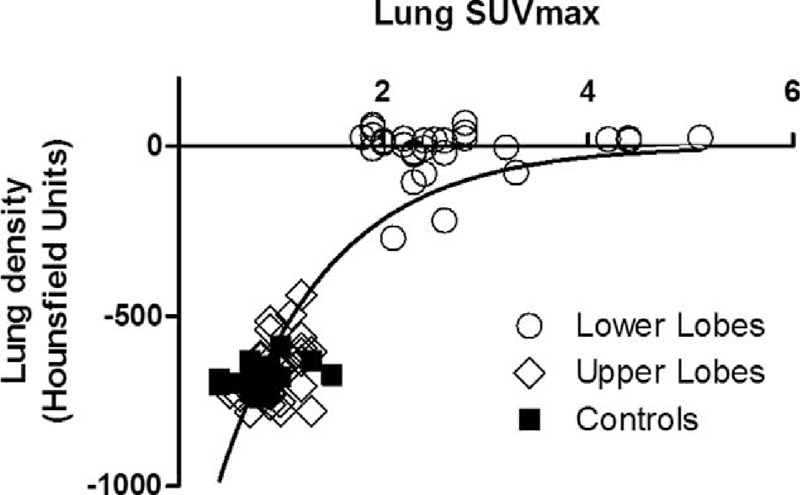
Lung density (HU) and maximal standardized uptake (SUVmax) values from ROIs of lower lobes (opened circles), upper lobes (opened diamonds), and sickle cell disease nonacute chest syndrome controls (closed squares). There was a significant correlation between lung density and SUVmax (lung density = −1436 × exp [−0.95 × SUVmax]; *R*^2^ = 0.78). Lower lobe ROIs exhibit HU values close to zero with scattered values of SUVmax, illustrating that changes in SUVmax values are not merely related to changes in lung density. ACS = acute chest syndrome, HU = Hounsfield units, ROIs = regions of interest, SCD = sickle cell disease.

Overall, there was a significant correlation between lung density and SUVmax (*R*^2^ = 0.78; Figure 5). ROIs with HU close to zero (ie, consolidated lung bases) exhibited scattered values of SUVmax, illustrating the intra-ROI heterogeneity of SUVmax and that SUVmax changes were not merely dependent from changes in lung density.

### Clinical Data

On ICU admission (Table 3), as well as on the day of PET/CT scan acquisition (Table 4), patients with SUVmax >2.5 were not significantly different from those with SUVmax ≤2.5 regarding most of the studied demographics, and clinical and laboratory features. The oxygen requirement was greater, with a trend toward higher arterial Pao_2_ levels and significantly lower Paco_2_ levels in patients with higher SUVmax as compared to others (Table 3). Patients with higher SUVmax also showed higher white blood cell counts and serum alanine transferase levels as compared to others (Table 3).

**TABLE 3:**
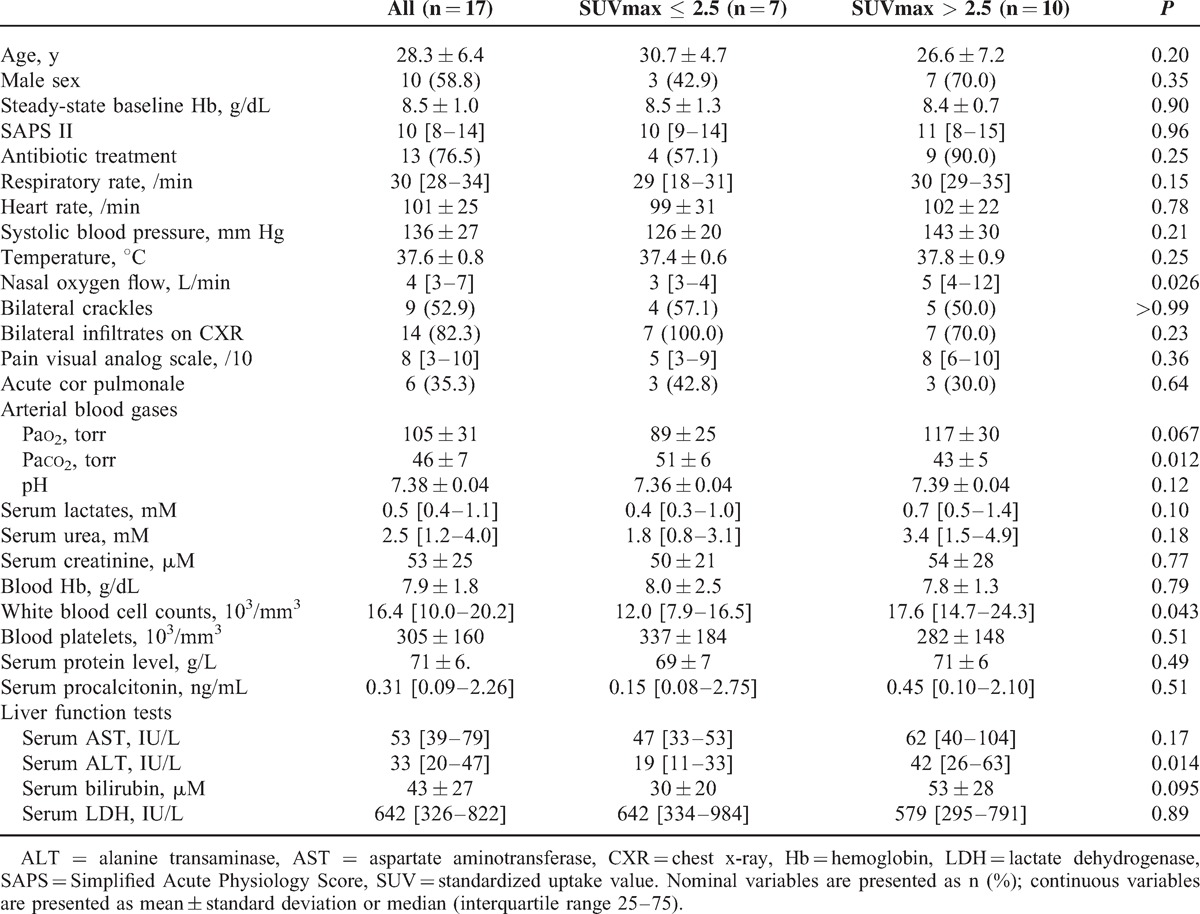
Demographics, Clinical Features, and Laboratory Test Values of Patients With Acute Chest Syndrome on Hospital Admission

**TABLE 4:**
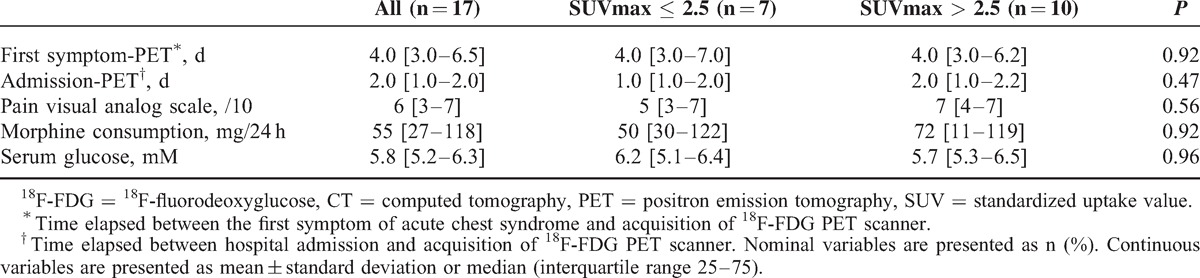
Characteristics of Patients With Acute Chest Syndrome on the Day of ^18^F-FDG PET Scan Acquisition

Three patients were diagnosed with a pulmonary infection (involving *Staphylococcus aureus*, coagulase-negative *Staphylococcus* and A/H1N1 influenza virus) and another patient had B19 parvovirus primary infection. The SUVmax of infected patients was not significantly different from that of noninfected patients (2.6 [2.2–2.8] vs 2.4 [2.1–3.0]; *P* = 0.55). None of the included patients died during hospital stay nor required invasive mechanical ventilation support. Biological features and clinical outcomes were similar between groups except for a significantly longer hospital stay in patients with SUVmax >2.5 (Table 5).

**TABLE 5:**
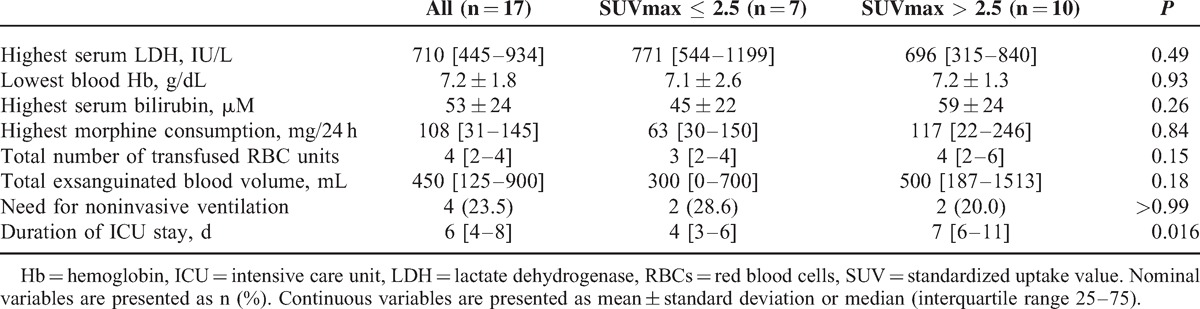
Laboratory Test Values Recorded During Intensive Care Unit Stay and Outcome of Patients With Acute Chest Syndrome

## DISCUSSION

The main findings of the current study are as follows: lungs of patients with ACS exhibited higher ^18^F-FDG uptake than SCD non-ACS controls, due to the presence of foci of hypermetabolism located in consolidated lung bases; lung apices were normally aerated and exhibited lower ^18^F-FDG uptake than lung bases, but higher ^18^F-FDG uptake than lungs of SCD non-ACS controls; there was a significant correlation between regional lung density and ^18^F-FDG uptake, but increases in lung metabolism did not merely result from increases in lung density; and ACS patients with higher lung metabolism (ie, SUVmax >2.5) had comparable presentation on admission, but required longer ICU stay than others.

### Interpretation of Lung ^18^F-FDG Uptake During ACS

As compared to other inflammatory nontumoral conditions and as quantified with SUVmax, lung ^18^F-FDG uptake of patients with ACS (median 2.5 [0.6–1.0]) was in the range of that reported in patients having lung contusion (2.2)^24^ or idiopathic pulmonary fibrosis (2.8),^23^ but lower than that reported in the pulmonary parenchyma of patients with sarcoidosis (5.1).^25^ Lung ^18^F-FDG uptake has been associated with lung neutrophilic inflammation in various conditions of acute lung injury, including experimental models of pneumococcal pneumonia,^9,26^ bleomycin pneumonitis,^9,26^ endotoxemia,^27,28^ ventilator-induced lung injury,^12^ smoke inhalation,^29^ and airway instillation of endotoxin in humans.^11^ Because increased bronchoalveolar fluid neutrophilia has been reported during ACS,^8^ one can hypothesize that neutrophils are the main cells accounting for lung ^18^F-FDG uptake during ACS. This hypothesis is consistent with our finding of increased white blood cell counts in patients with lung SUVmax >2.5, as compared with others. Nevertheless, the contribution of other inflammatory cells such as alveolar macrophages, monocytes, or lymphocytes to lung ^18^F-FDG uptake cannot be ruled out, as previously suggested by neutrophil depletion experiments^12^ and further illustrated by microautoradiographic studies in other settings.^30^ For instance, the role of endothelial cells, known to be highly metabolically active and to take up ^18^F-FDG after nitric oxide stimulation,^31,32^ a key mediator in the pathophysiology of sickle cell vasoocclusion phenomena,^33^ would need to be explored in further studies in order to better understand the mechanisms of lung ^18^F-FDG uptake during ACS.^23–25^

### Heterogeneous Increase in Lung ^18^F-FDG Uptake During ACS

The current study shows increased ^18^F-FDG uptake in lungs of ACS patients, as compared to SCD non-ACS controls, with heterogeneous distribution. Consolidated lung bases exhibited dramatically higher metabolism than normally aerated apices. The mechanisms accounting for the predominant location of consolidated regions in lung bases during ACS have been previously discussed,^7^ and may include the effect of gravity, amplified by increased lung weight related to permeability-type lung edema and subsequent pressure gradient along the ventrodorsal axis; the diaphragmatic cranial displacement, due to increased abdominal pressure; and the superimposed pressure of heart weight on lung tissue. In our series, increased lung densities mainly resulted from regional lung consolidation probably associated with alveolar inflammation and infiltration by plasma-like edema fluid, as suggested by higher ^18^F-FDG uptake. Such heterogeneity in lung ^18^F-FDG uptake, following a ventrodorsal gradient with higher cellular metabolic activation in lung bases than in apices, has been previously reported in experimental,^27,28,34^ as well as in clinical acute respiratory distress syndrome,^15^ and was mainly attributed to mechanical forces associated with ventilator-induced lung injury. In the current series, including no mechanically ventilated patients, the higher ^18^F-FDG uptake in lung bases could, at least partially, be perfusion-related, lung bases exhibiting more regional perfusion than apices.^35^ The effect of lung regional perfusion distribution on ^18^F-FDG uptake is debated^24,27,34^; yet, a positive relationship between perfusion and ^18^F-FDG uptake was demonstrated in an experimental model of acute lung injury and endotoxemia,^27^ likely related not only to increased regional load of inflammatory cells and mediators in regions receiving more perfusion, but also to higher endotoxin delivery in these regions leading to more regional inflammation. During ACS, lung injury is theoretically driven by a vascular insult, and a positive relationship between regional perfusion and ^18^F-FDG uptake would be expected.

Previous studies in patients with lung contusion^16^ and the acute respiratory distress syndrome^15^ showed the ability of ^18^F-FDG PET to detect increased metabolism in regions with normal lung aeration. As compared to lung density changes detected by conventional x-ray imaging, ^18^F-FDG uptake may serve as an early marker of lung inflammation during acute lung injury. In line with these findings, in the current study, normally aerated apical lung regions of ACS patients showed higher ^18^F-FDG uptake than those of SCD non-ACS controls, suggesting that normally aerated regions might also be affected by early inflammatory phenomena, which are detectable only with ^18^F-FDG PET. As control patients were selected among a cohort of SCD patients followed-up in our institution, increased ^18^F-FDG uptake in these normally aerated regions cannot be ascribed to changes in pulmonary metabolism related to sickle cell anemia itself, but would rather be related to manifestations of ACS leading to hypermetabolism in the tomodensitometrically healthy lung, possibly involving vasoocclusion phenomena in the pulmonary microvasculature or pulmonary blood flow redistribution toward the upper lobe resulting from hypoxic vasoconstriction in consolidated lower lobes. Nevertheless, the pathophysiologic significance of the increased ^18^F-FDG uptake in normally aerated regions during ACS remains unclear and needs further studies.

Changes in regional lung density can artificially increase ^18^F-FDG uptake measurements when lung density changes are related to loss of aeration, as opposed to lung infiltration by metabolically active cells (eg, neutrophils).^13^ In this study, the fact that lower lobe ROIs with HU values close to zero had scattered values of SUVmax (Figure 5) suggests that changes in ^18^F-FDG uptake in consolidated regions were not merely related to changes in lung density, as would occur in purely atelectatic lung regions. This is consistent with the review of CT scans by a radiologist blinded to the interpretation of ^18^F-FDG PET scans who confirmed that atelectasis was present in a minority of segments (Table 1), and thus that changes in regional ^18^F-FDG uptake would not be a direct consequence of loss of aeration.

### Clinical Implications

The comparison of patients with higher versus lower lung metabolism showed similar findings in terms of clinical presentation, pain intensity, morphine requirement, need for RBC transfusions, and markers of hemolysis. In fact, our patients were very homogeneous in terms of presentation and outcomes; none died nor required invasive mechanical ventilation, thereby limiting the ability to show differences between groups of patients exhibiting different degrees of lung metabolism. Intriguingly, patients with SUVmax >2.5 exhibited significantly lower Paco_2_ upon admission than others, reminiscent of a previous series in which ACS patients having acute pulmonary hypertension and poorer outcome were less hypercapnic.^18^ Some patients with severe ACS exhibit hypercapnia, probably reflecting hypoventilation related to chest pain and/or opioids. Conversely, normocapnia during ACS may be associated with more extensive lung parenchyma involvement and higher severity of disease, as reflected by pulmonary hypertension and lung inflammation. Patients with SUVmax >2.5 also had longer ICU stay than others, consistent with a more severe disease, with more intense pain and longer delay in hypoxia resolution. The predictive value of ^18^F-FDG PET during ACS, and the clinical relevance of the findings from this imaging tool in this setting, would need to be confirmed in a larger series of patients including more severe ACS.

### Limitations of the Study

Our study has several limitations. First, this study included a limited number of patients from a single center, with homogeneous presentation and outcomes, thereby limiting the generalizability of results and the ability to show outcome differences. Indeed, the most severe patients (ie, those requiring mechanical ventilation) were excluded from this study because transport to the PET/CT facility was considered hazardous. Second, ^18^F-FDG uptake was quantified using SUV, a static index that does not allow for studying ^18^F-FDG kinetics parameters, only available with dynamic indexes.^13^ Still, using a static index allows for reducing the time of acquisition, thereby limiting the time spent by patients out of the ICU, and acquiring images over many bed positions in order to scan the entire lungs. Third, using SUV did not allow for correcting ^18^F-FDG uptake for regional lung density, as previously performed with dynamic indexes.^12,13,15,27,36^ Fourth, as discussed earlier, the mechanisms involved in the higher metabolism observed in upper lung regions of ACS patients as compared to SCD non-ACS controls are unclear. Obtaining an additional control group including ICU patients with SCD but no ACS might have allowed discriminating whether such higher lung metabolism is related to the ACS process itself or to systemic manifestations associated with severe SCD.

## CONCLUSIONS

Lungs of patients with ACS exhibit higher ^18^F-FDG uptake, with foci of hypermetabolism located in consolidated lung bases. Lung apices had normal aeration and lower ^18^F-FDG uptake than lung bases, but higher ^18^F-FDG uptake than lungs of SCD non-ACS controls, suggesting early inflammation in these regions, and a larger involvement of the lung during ACS than indicated by conventional imaging.
